# FallFitness exercise program provided using the train-the-trainer approach for community-dwelling older adults: a randomized controlled trial

**DOI:** 10.1186/s12877-024-05575-0

**Published:** 2024-11-30

**Authors:** Marina Arkkukangas, Karin Strömqvist Bååthe, Julia Hamilton, Ali Hassan, Michail Tonkonogi

**Affiliations:** 1 Department of Medicine, School of Health and Welfare, Dalarna University, Falun, Sweden; 2 Department of Physiotherapy, School of Health, Care and Social Welfare, Mälardalen University, Västerås, Sweden; 3 Research and Development Department, Region Sörmland, Eskilstuna, Sweden; 4 Sabbatsbergs Department of Geriatric Medicine, Region Stockholm, Stockholm, Sweden

**Keywords:** Falls, Falling techniques, Older adults, Public health

## Abstract

**Background:**

Falls and fall-related injuries remain a global challenge and threat to the health of older adults. Specific strength and balance exercises are effective in preventing falls among community-dwelling older adults. Nevertheless, provision of evidence-based fall prevention interventions to a broad population represents a healthcare challenge, indicating that new models for promoting exercise among community-dwelling older adults need to be addressed. Here, we aimed to evaluate the effects of a peer-led group-based exercise intervention provided using the train-the-trainer approach and targeting physical performance, activity level, handgrip strength, quality of life, fall-related self-efficacy, fear of falling, and falling techniques compared with a control group at 8-week follow-up.

**Methods:**

This randomized controlled trial (RCT) included trainers and participants who were recruited from four collaborating regional organizations for retired persons. The intervention was planned to be provided in five municipalities in Sweden, depending on the location of the registered trainers.

Eligible participants included adults aged ≥ 60 years who could walk independently and understand written and oral information in Swedish. The FallFitness multicomponent exercise program delivered weekly strength, balance, and falling techniques over eight weeks. It was evaluated using the train-the-trainer approach. Fourteen older adults were eligible for trainer education, and 101 participants were randomly allocated for the FallFitness exercise (*n* = 50) or a control group (*n* = 51).

**Results:**

After 8 weeks of peer-led training, the short multicomponent exercise program significantly improved the physical activity levels (*p* = 0.036) and backward and sideways falling techniques (*p* < 0.001) compared to those in the control group. Fear of falling significantly decreased in the exercise group (*p* = 0.009). Other outcomes in this study showed to be non-significant.

**Conclusions:**

The multicomponent exercise program provided in eight sessions using the train-the-trainer approach may be effective in promoting physical activity and the learning of motor skills and safe landing strategies. Furthermore, the FallFitness exercise program may reduce the fear of falling and may be both time- and cost-effective.

**Trial registration:**

ClinicalTrials.gov, NCT06265480 (20240208).

## Key points

•Older adults can be educated about learning motor skills and safe landing strategies and transfer such knowledge to their peers.

•The group-based multicomponent exercise program improved the physical activity levels in community-dwelling older adults.

•Participation in the short multicomponent exercise program also reduced their fear of falling.

•The train-the-trainer approach may empower older adults to advocate fall prevention measures in their communities.

•This approach enables early and accessible primary fall prevention among community-dwelling older adults beyond healthcare responsibilities.

## Background

Evidence supporting exercise as the most effective intervention for reducing falls in community-dwelling older individuals is well established [1]. In particular, exercises tailored to enhance balance and functional capacity have pronounced effects in preventing falls. Findings from the 2019 Cochrane Review focusing on older adults living independently have confirmed that exercise intervention significantly reduces the occurrence of falls, with balance and functional exercises contributing to a 24% reduction in the fall rate [2, 3]. Moreover, programs incorporating a combination of balance, functional, and resistance exercises exhibit even greater efficacy, reducing the fall rate by 34% [3].

Evidence supporting exercise as a preventive measure is therefore stronger for independently living older adults than, for example, in residential care settings [4]. Other previous reports also pointed out that exercise programs could be successful if they were structured, supervised, and resourced to deliver an adequate dose of training [5, 6]. While previous studies have shown that exercise reduces falls, there is a need for exercises specifically aimed at reducing fall-related injuries. Haagsma et al. reported that 11.7 million adults aged ≥ 70 years in Western Europe sought medical attention for injuries in 2017; among them, 8.4 million (71.9%) experienced falls, with the rate of fall-related injuries requiring healthcare being higher in women than in men [7].

To address fall-related injuries, recent studies have suggested the incorporation of complementary falling techniques into fall prevention programs [8, 9]. Falling techniques may reduce the bone impact force and velocity when falling naturally. However, this area requires further investigation, as few studies have evaluated falling techniques and their impact on fall-related injuries [10]. Therefore, further investigation on falling techniques and on whether they can be learned and performed by older adults is of great importance.

Interventions for fall prevention should be introduced early in life, as indicated by global recommendations for fall prevention, which suggest opportunistic case finding and proactive engagement with healthcare providers through inquiries about falls [1]. Unfortunately, early preventive measures are often overlooked within healthcare systems [11–13], leading to scenarios in which exercises for fall prevention are initiated only after a fall has already occurred. This delay in intervention underscores the necessity for a paradigm shift towards more proactive approaches to fall prevention, ensuring that individuals receive appropriate guidance and exercise intervention to reduce the risk of falls before incidents occur.

Healthcare professionals face considerable constraints in proactively addressing fall prevention, and collaboration with other stakeholders is required to develop and implement early interventions. Previous studies exploring the perspectives of healthcare professionals regarding fall prevention have identified various barriers, including time constraints, competing priorities, financial limitations, and knowledge gaps [13, 14]. Owing to these obstacles, a significant proportion of patients at risk for falls or with a history of falls frequently do not receive adequate fall prevention care.

The availability of fall prevention measures can be extended and increased through the train-the-trainer approach. Recently, in 2021, Logan et al. employed this method to educate all staff in participating care homes and reported a reduction in falls by 43% [15]. Furthermore, other previous studies obtained promising results in terms of strength, balance, and decreased fall incidence when older adults were educated about peer-led fall prevention exercises [16, 17].

Considering the challenges in fall prevention among community-dwelling older adults, a project using the train-the-trainer approach was launched in Sweden in 2023. As part of their education, participants were trained in the FallFitness exercise program, a newly developed program designed to improve balance, strength, and falling techniques and inspired by the previously developed Judo4Balance program [8].

The present study aimed to evaluate the effects of a peer-led group-based exercise intervention provided using the train-the-trainer approach and targeting physical performance, activity level, handgrip strength, quality of life, fall-related self-efficacy, fear of falling, and falling techniques compared with a control group at 8-week follow-up.

## Methods

This randomized controlled trial (RCT) sought to include 100 participants: 50 participants were randomly assigned to the exercise group and 50 participants to the control group. This RCT was reported in accordance with the Consolidated Standards of Reporting Trials (CONSORT) checklist and was registered at ClinicalTrials.gov (NCT06265480). In this study, the 8-week follow-up was reported. Future follow-ups on fall frequency would occur at 12 months after baseline measurements and were therefore not reported in this study.

### Participants and setting

For this study, trainers and participants were recruited from four collaborating regional organizations for retired persons within a small region in middle Sweden. Details about the study were provided in a specific meeting with the research group, which was advertised by the collaborating organizations. Verbal and written information was provided to those who showed an interest in participating. Oral and written informed consent was obtained from the participants during baseline measurements. The intervention was thereafter planned to be provided in five municipalities, depending on the location of the registered participants.

The eligibility criteria for the trainers and 100 participants in the intervention were as follows: adults aged ≥ 60 years who could walk independently and understand written and oral information in Swedish. The exclusion criteria were as follows: individuals who were too physically weak to sit upright without support, who were unable to hold their neck up when lying or rolling backward, who had a history of aortic aneurysm or unstable angina, and who had recently undergone cataract surgery.

### Intervention

Recruitment was performed in two steps. First, older adults who met study criteria and showed interest in exercises and the FallFitness exercise program were invited to participate, aiming for a minimum of 10 trainers. Second, participants who showed interest in the intervention were invited for baseline measurements and then randomized to the FallFitness exercise group or control group, aiming for a total of 100 participants. The trainers were not included in the baseline or follow-up measurements.

#### Train-the-trainer education

The train-the-trainer approach was used in this study. This method involved recruiting volunteer older adults from pensioner organizations and providing them with comprehensive education about fall prevention by professionals with solid knowledge on fall prevention, aging, exercise, and judo-inspired falling techniques. The education of these trainers consisted of 3 days of theory, in which they were provided with literature on fall prevention and exercise, and practical training in balance, strength, and fall prevention exercises. The educators had extensive experience in leading group exercises for older adults. The theoretical part of the education was led by a physiotherapist with extensive experience working with older adults and fall prevention research, alongside a professor of medical science. For the practical part of the education, a physiotherapist, with a black belt in judo, specialized in senior health with long experience of working with older adults, along with a black belt judo instructor with a coach education license, were involved. The recruited trainers worked in pairs to prepare for the group-based exercise intervention evaluated in this study. During the 8-week intervention, trainers were offered a 1-h consultation per week to support them and discuss any type of challenges that might have emerged during their sessions. Additionally, they received a handbook with a detailed explanation of the program setup, and each lesson (a total of 8 sessions) was described exercise by exercise. When a new exercise was introduced in the lesson, it was highlighted to clearly show progression. Each trainer was instructed to follow a lesson plan. The trainers at the five locations were responsible for inviting the participants who were eligible and randomized to receive the intervention. The trainers had a protocol for attendance, which they reported to the first author.

#### FallFitness exercise program

The exercise program comprised 60-min training sessions that were offered once a week for 8 consecutive weeks, in five groups with ten participants in each. Each session included warm-up, balance and functional strength training, falling techniques, and cooldown. The sessions had a similar structure with logical progression from easy to hard, as well as simple to more complex exercises. These exercises were introduced successively throughout the 8-week period. The intensity level of the training program was moderate.

The FallFitness exercise program consisted of two main blocks: Block 1 was a 4-week period that mainly focused on adaptation to training. Each session included strength exercises targeting large muscle groups, such as push-ups, chair rises, toe raises, and pelvic lifts. Participants completed two sets of 10–12 repetitions for each exercise. Modifications were available to accommodate individual fitness levels. For instance, participants could perform push-ups against a wall, on their knees, or as standard push-ups. The sessions also included simple balance exercises, static activities, such as standing with feet together or in a tandem position and standing on one leg. More dynamic exercises involve shifting weight from one leg to the other and walking along a line using tandem steps, both forwards and backwards, reaching exercise and even exercises based on the dual task. Further, getting up and down from the floor, and backward and sideways falling techniques from a sitting position were included.

Block 2 was an additional 4-week period that focused on working in pairs, dual tasks, multicomponent balance exercises, reactive balance training, more complex backward and sideways falling techniques from squatting and standing positions, and “power strength” exercises.

#### Control group

The control group participated in baseline and 8-week follow-up measurements and received no specific treatment during this period. However, they were invited to participate in the FallFitness exercise program after all fall calendars were completed at 12 months after baseline measurements.

### Measurements

Two independent assessors performed single-blind measurements before randomization and at 8 weeks after randomization. The assessors were well familiar and experienced in using all measurements performed at baseline and at eight- week follow-ups. One physiotherapist and one judo instructor performed all measurements. The setting was the facility in each municipality where the exercise was planned to be performed.

Data on participants’ demographics and self-rated secondary outcomes, quality of life, fall-related self-efficacy, and fear of falling were obtained through a questionnaire. Additionally, participants were instructed to complete a printed fall calendar monthly for 12 months. The primary outcome was physical performance, whereas the secondary outcomes were activity level, handgrip strength, and falling technique. The physical performance in the lower extremities was assessed using the Short Physical Performance Battery (SPPB). SPPB includes strength, balance and walking items and the scale scores range from 0 to 12 points, a total score of 12 represents the best performance [18]. Physical activity was evaluated using the Frändin–Grimby Activity Scale [19]. The scale is well used in both research and practice and assesses leisure-time physical activity, including household activities. The scale ranges from 1 to 6 with 1 representing “hardly active at all” and 6 representing “intensive exercise regularly and several times per week”. Handgrip muscle strength was measured using a Jamar hand dynamometer to provide a valid measure of general body strength [20]. Health-related quality of life and its five dimensions (mobility, self-care, usual activities, pain, and anxiety/depression) were assessed using the EQ-5D and EQ-5D VAS [21]. The level of concern regarding falling was determined using the Falls Efficacy Scale-International, which is a short, easy-to-administer tool that measures the level of concern about falling during 16 social and physical activities inside and outside the home, irrespective of whether the person actually performed the activity [22]. The fear of falling was evaluated using the Fear of Falling Questionnaire-Revised (FFQ-R), which is a 15-item tool for measuring the fear of falling [23]. The FFQ-R was translated into Swedish and subjected to a psychometric evaluation by the research team. The judo-inspired backward and sideways falling techniques were graded on a 0–4 scale using the Strömqvist Bååthe Falling Competence Test [24].

### Data analysis

#### Power calculation and randomization

A stratified randomization was performed to allocate participants into exercise and control groups. A total of 101 participants were stratified into five equal groups based on their municipalities in region. Randomization within each stratum was conducted using Microsoft Excel, where the RAND function generated random numbers for each participant. Participants were then sorted based on these random numbers, with 50 participants assigned to the exercise group and 51 to the control group. A statistician performed the randomization, and the first author informed the trainers about the participants allocated to the exercise and control groups. The research team had previously conducted a similar program and calculated the sample size based on the results of that study. We believe that the intensity might decrease when not conducted by a licensed judo instructor. Therefore, we hypothetically calculated that a clinically relevant difference of 1 point (standard deviation [SD]: 1.5) in the SPPB would require 36 participants in each group, assuming a power of 80% and alpha of 0.05. However, due to the uncertainty of using the train-the-trainer approach, we decided to include 102 participants in the study because we had access to 102 participants.

#### Statistical methods

Descriptive statistics, including the mean, standard deviation (SD), median, minimum, and maximum, were calculated. Non-parametric methods were used for all tests due to the ordinal nature of the data. Differences between the exercise and control groups were analyzed using the Mann-Whitney U test (for two independent samples).

The Wilcoxon signed-rank test was used to assess differences between baseline and 8-week follow-up values within each group for paired samples. Two-tailed p-values were calculated, with statistical significance set at *p* < 0.05. The Last Observation Carried Forward (LOCF) method was used to handle missing data, ensuring that the analysis adhered to the Intention To Treat (ITT) principle. Sensitivity analyses were conducted to assess the impact of dropouts on study outcomes. All analyses were conducted using SPSS version 29.0 (IBM Corp. Armonk, NY, USA) on Windows.

### Ethical considerations

The participants received no compensation for their participation in the study. Written informed consent was received from all participants. The current study was approved by the Swedish Ethical Review Authority (approval no. 2023–04577-01) and was conducted in accordance with the principles embodied in the 1964 Declaration of Helsinki, which concerns human rights, informed consent, and correct procedures regarding treatment in research involving human participants.

## Results

A total of 102 community-dwelling older adults from five municipalities in central Sweden who lived in their own homes were deemed to be eligible to participate in this study; however, one adult declined to participate prior to baseline measurements (Table 1). The recruitment, intervention, and follow-up period was from February to June 2024.

**Table 1:** Baseline characteristics of participants

Characteristics	Total (*n* = 101)	Exercise (*n* = 50)	Control (*n* = 51)
Age (mean, SD)	75.8 (5.7)	75.3 (5.6)	76.4 (4.4)
Sex (female/male)	84/17 (83.2/16.8)	42/8 (84/16)	42/9 (82.4/17.6)
Highest level of education (*n* = 100)			
Elementary or junior secondary high/girls’ school	22 (22)	9 (18.4)	13 (26.5)
Upper secondary school or university/college	78 (78)	40 (81.6)	38 (73.5)
Housing and living arrangement (*n* = 100)			
Apartment block	52 (52)	22 (44.9)	30 (58.8)
Townhouse or villa	48 (48)	27 (55.1)	21 (41.2)
Living alone/living with someone (*n*=100)	48/52 (48/52)	26/23 (53.1/46.9)	22/29 (43.1/56.9)
Assistance with daily activities (*n* = 100)			
No	95 (95)	45 (91.8)	50 (98)
Yes, from relatives	1 (1)	1 (1)	0 (0)
Yes, from a private company (ex. cleaning company)	4 (4)	3 (6.1)	1 (2)
Walking aid (*n* = 100), possible to choose more than one option to answer it			
No	83 (83.8)	39 (79.6)	44 (88)
Yes, outdoors	16 (16)	10 (20,4)	6 (12)
Yes, indoors	1 (1)	1 (2)	0 (0)
Falls during the year before baseline (*n* = 99)			
No	51 (51.5)	25 (50)	26 (53.1)
Yes, one fall	34 (34.3)	15 (30)	19 (38.8)
Yes, two or more falls	14 (14.2)	10 (20)	4 (8.2)

Values are presented as numbers (%), *SD* standard deviation

**Fig. 1:**
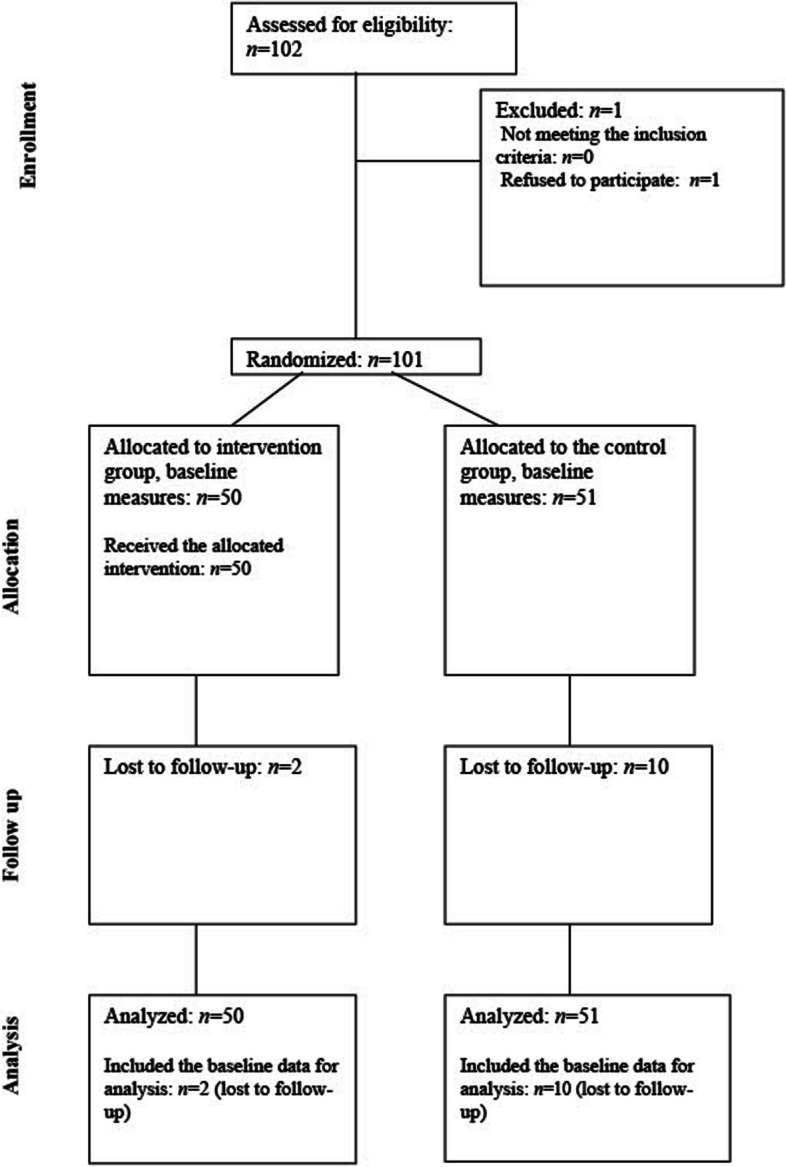
Flowchart of the trial

Table 1 summarizes the participants’ characteristics, and Fig. 1 presents the flowchart of the trial. Participants’ mean age was 76 years. Half of the participants experienced one or more falls during the last year. Descriptive comparisons between the groups at baseline and at the 8-week follow-up are shown in Table 2. Out of 14 eligible trainers, 13 completed the 3-day education program; 10 women and 3 men were licensed as FallFitness exercise program instructors. At baseline, no significant differences were found between the groups in any of the measures.

**Table 2:** Descriptive comparison between baseline and 8-week follow-up

	Baseline		8-week follow-up	
	Exercise group	Control group	Exercise group	Control group
SPPB (0–12), median (min–max)	11.5 (5–12)	11 (1–12)	12 (7–12)	11 (1–12)
Handgrip, average left/right (kg), mean (SD)	23.5 (7.5)	23.6 (6.7)	24.3 (7.6)	23.3 (6.9)
EQ-5D TTO, median (min–max)	0.94 (0.78–0.97)	0.94 (0.71–0.97)	0.93 (0.59–0.97)	0.93 (0.71–0.97)
Rated health status (0–100), median (min–max)	77.5 (30–100)	80 (20–100)	80 (30–100)	80 (30–100)
FFQ–R-S (15–60), median (min–max)	33 (22–48)	32.5 (24–47)	31 (21–42)	32 (21–48)
FES-I (16–64), median (min–max)	20 (16–41)	22 (16–38)	18 (16–36)	19 (16–38)
Frändin–Grimby (1–6), median (min–max)	4 (2–5)	3 (0–6)	4 (1–5)	3 (1–5)
Fall competence backward score (0–4), median (min–max)	1 (0–4)	1 (0–2)	2 (0–4)	1 (0–3)
Fall competence sideways score (0–4), median (min–max)	1 (0–2)	1 (0–2)	2 (0–4)	1 (0–2)

*SPPB* Short Physical Performance Battery, *EQ-5D* EuroQol-5D-5 L, *TTO* Time Trade Off, *FFQ-R-S* Fear of Falling Questionnaire-Revised- Swedish version, *FES-I* Falls Efficacy Scale-International

For the within group analysis the Wilcoxon signed-rank test was used to assess within-group differences between baseline and the 8-week follow-up for measures including SPPB, handgrip strength, EQ-5D TTO, rated health status, FFQ-R-S, FES-I, Frändin-Grimby Activity Level Scale, fall competence backward score, and fall competence sideways score. The results for each group are presented in Table 3. Significant improvements were observed in the exercise group at the 8-week follow-up for FFQ-R-S (*p* = 0.01), fall competence backward score (*p* < 0.001), and fall competence sideways score (*p* < 0.001).

**Table 3:** Descriptive statistics at the 8-Week Follow-Up and within group analysis between baseline and 8-week Follow-Up

	Exercise group	*p*-value	Control group	*p*-value
SPPB (0–12), median (min–max)	12 (7–12)	0.170	11 (1–12)	1.00
Handgrip, average left/right (kg), mean (SD)	24.3 (7.9)	0.08	23.3 (6.9)	0.871
EQ-5D TTO, median (min–max)	0.93 (0.59–0.97)	0.209	0.93 (0.71–0.97)	0.700
Rated health status (0–100), median (min–max)	80 (30–100)	0.134	80 (30–100)	0.085
FFQ-R-S (15–60), median (min–max)	31 (21–42)	**0.009**	32 (21–48)	0.486
FES-I (16–64), median (min–max)	18 (16–36)	0.259	19 (16–38)	0.710
Frändin–Grimby (1–6), median (min–max)	4 (1–5)	0.835	3 (1–5)	0.815
Fall competence backward score (0–4), median (min–max)	2 (0–4)	**< 0.001**	1 (0–3)	0.688
Fall competence sideways score (0–4), median (min–max)	2 (0–4)	**< 0.001**	1 (0–2)	0.688

*SPPB* Short Physical Performance Battery, *EQ-5D* EuroQol-5D-5 L, *TTO* Time Trade Off, *FFQ-R-S* Fear of Falling Questionnaire-Revised- Swedish version, *FES-I* Falls Efficacy Scale-International. Significant results are highlighted in bold.

For the between group analysis the Mann-Whitney U test (for two independent samples) was used to assess the differences between the exercise and control groups at the 8-week follow-up for measures including the SPPB, handgrip strength, EQ-5D TTO, rated health status, FFQ-R-S, FES-I, Frändin-Grimby Activity Level Scale, fall competence backward score, and fall competence sideways score, as shown in Table 4. The test results showed a significant difference between the groups on the Frändin-Grimby Activity Level Scale (*p* = 0.036) and in both the falling backward and falling sideways techniques (*p* < 0.001), as detailed in Table 4.

**Table 4:** Between the groups analysis at 8-week Follow-Up (independent sample Mann-Whitney U test)

	*p*-Value	Effect-size (*r*)
SPPB (0–12)	0.205	0.13
Handgrip, average left /right(kg)	0.606	0.05
EQ-5D TTO	0.805	0,02
Rated health status (0–100)	0.579	0.05
FFQ-R-S (15–60)	0.222	0.12
FES-I (16–64)	0.735	0.03
Frändin-Grimby (1–6)	**0.036**	**0.21**
Fall competence backward score (0–4)	**< 0.001**	**0.48**
Fall competence sideways score (0–4)	**< 0.001**	**0.55**

*SPPB* Short Physical Performance Battery, *EQ-5D* EuroQol-5D-5 L, *TTO* Time Trade Off, *FFQ-R-S* Fear of Falling Questionnaire-Revised- Swedish version, *FES-I* Falls Efficacy Scale-International. Significant results are highlighted in bold.

The participants in the training group participated in the training session with the trainer seven out of eight times on average (73%), with a minimum and maximum individual participation of four and eight, respectively. No adverse advents were reported during the intervention.

## Discussion

This RCT showed that the short multicomponent FallFitness exercise program provided using the train-the-trainer approach significantly improved physical activity levels and backward and sideways falling techniques compared with those in the control group after 8 weeks of peer-led training. Our observations suggest that motor skills and safe landing strategies could be learned within a short period, and that the FallFitness exercise program may promote improved physical activity levels. Group-based exercises lead to increased physical activity [25], which is a positive outcome, as highlighted in a recent study published in Lancet and supported by the World Health Organization [26]. Additionally, the results indicated a significant reduction in the fear of falling in the exercise group. This is valuable knowledge because the fear of falling is associated with a high risk of future falls and functional decline [27, 28].

The findings of our study constitute an important addition to the field, as recent research on fall prevention has emphasized that effective fall prevention exercises are typically supervised by professional trainers or healthcare professionals [29]. From an economic perspective, supervised programs have greater monetary value than unsupervised programs [30]. By using the train-the-trainer approach, future demographic challenges may be addressed, and collaboration between civil society and healthcare can be strengthened because the model is based on experts educating older adults to become trainers. This information can be used for planning and implementing future programs or future models investigating the best monetary value of such programs and are also in line with the PROFANE taxonomy which emphasizes self-management and empowering older adults with an active role in fall prevention [31].

In this RCT, we explored new primary prevention methods beyond traditional sports and wellness organizations and healthcare responsibilities/possibilities by using the train-the-trainer approach, implementing a short 8-week program, and including falling techniques in the fall prevention program [11, 32]. In this study, older adults with a wide range of backgrounds and experience in leading exercise groups (from novice to experienced individuals) were recruited. None of the trainers had previous backgrounds in martial arts, where the use of breakfall techniques is common, in this specific population of older adults [33]. Despite a relatively short period of training for breakfall skills, these skills seem transferable to the exercise group. Education about fall prevention is frequently used by healthcare professionals in fall prevention care [34] and, to our knowledge, is less common among older adults when integrating theory and practice.

Attendance should be emphasized, as an adherence rate above 80% may significantly reduce the risk of falls compared with lower adherence rates in community-dwelling older adults [35]. Group-based exercise interventions in the same population as in our study have shown an adherence rate of 69.1% [36]. In this study, an attendance rate of 73% was observed for participants attending seven out of eight sessions, reflecting the train-the-trainer approach as a successful model for participation in the exercise, as well as the geographical convenience of the location of the exercise group, which is in line with previous research in the field [36].

The results of this study indicate that a relatively short training period (i.e., 8 weeks with one training session per week) could be a relatively time- and cost-efficient fall prevention intervention. This approach may also contribute to increased physical activity, which was observed to have a positive effect in the present study. Further, our study found significant improvements in falling techniques following 8-week training program, demonstrating that substantial advances in motor skills in this age group can be achieved within the same time frame as improvements in muscle strength and function. American College of Sports Medicine guidelines recommend a minimum duration of 6 weeks for noticeable improvements in strength due to increased neurological efficiency [37]. Furthermore, an 8-week period is considered the minimum time required for significant physiological changes in muscle structure and strength [38, 39].

A previous study by our team showed that falling techniques could be learned over 6–9 weeks and maintained in the long term under the supervision of a licensed judo instructor [9]. The implementation of the train-the-trainer approach to educate older adults on providing exercise intervention addresses crucial factors for primary prevention, such as availability and implementation for a broader range of older adults. Considering the slow and limited implementation of fall prevention programs in practice [12, 40], new ways of addressing successful implementation must be prioritized in the future. Ongoing research is investigating the implementation of fall prevention guidelines in Norway [41]; nonetheless, the use of older adults as primary fall prevention providers remains sparsely investigated.

### Limitations and strengths

This study had some limitations. The primary outcome calculated on the SPPB raised some discussion in our research team regarding whether the SPPB was suitable to use for power calculation in this sample of active older adults, with a median value of 11 out of 12 points, suggesting a ceiling effect when used in higher-functioning older adults [42]. Furthermore, this study focused on physical function as the primary outcome and not falls. However, we consider our primary and secondary outcomes as important risk factors for falls, as underscored by numerous studies on fall prevention [1, 3].

The decision to perform an ITT analysis and increased risk of type II errors when conducting an ITT analysis must be addressed [43]. An ITT analysis is commonly performed in RCTs, and we judged that this was appropriate in our study because of the homogeneity in the sample. As dropout rates in both the intervention and control groups were judged to be low, they are unlikely to have affected the results.

Treatment fidelity was judged as a strength of this study, with thoroughly followed protocols, weekly meetings for trainers to attain standardization of the FallFitness exercise program, and opportunities to jointly discuss feasibility.

## Conclusions

This study showed that the short multicomponent FallFitness exercise program provided using the train-the-trainer approach significantly improved physical activity levels, which are crucial for counteracting inactivity in community-dwelling older adults. Furthermore, we observed that motor skills and safe landing strategies could be learned within a short period, which is important for preventing fall-related injuries. Participation in the FallFitness exercise program also significantly reduced the fear of falling, which is a major risk factor for both initial and future falls.

This initiative can empower older adults to become advocates for fall prevention within their communities while simultaneously offering beneficial exercise interventions tailored to reduce falls and related injuries. By utilizing the train-the-trainer approach, this program may also be time- and cost-effective and may reduce the burden on healthcare services. However, its impact on falls and fall-related injuries, as well as the burden on healthcare, needs to be further investigated in future studies.

## Data Availability

Aggregated data that support the findings of this study are available on reasonable request from the corresponding author, MA.

## Abbreviations

FES-I: Falls Efficacy Scale-International
FFQ-R: Fear of Falling Questionnaire-Revised
FFQ-R-S: Fear of Falling Questionnaire-Revised- Swedish version
ITT: Intention-To-Treat
RCT: randomized controlled trial
SD: standard deviation
SPPB: Short Physical Performance Battery
TTO: Time Trade =Off
